# Annotations

**Published:** 1887-03-12

**Authors:** 


### ANNOTATIONS.

Newcastle leads the Van.

PRACTICAL benevolence commends itself to the instincts and judgment of practical people. The Novocastrians are a practical race, and have taken the business of their infirmary in hand like men. Half measures have found no favour with them. Having begun a good work, they have determined to make it complete. A few weeks ago we were able to present a report, prepared by a Special Committee of the Newcastle Infirmary, which for extent of survey, fulness of detail, and wisdom of suggestion could hardly be surpassed. Pushing forward their work on the lines laid down in that report, the managers of the institution have now determined upon the bold course of putting an end to the special privileges of ordinary subscribers, and making their institution in the fullest sense of the word "a free hospital." For the future governors' letters will be things unknown. The principles of selection will be priority of application and urgency of need—"first come first served." Except where circumstances compel some other course, this should be the established rule at all public hospitals. It is satisfactory to note that special attention is called to the expenditure on stimulants, with a view to some diminution in that direction. A great deal too much is spent on alcohol at most hospitals. Institutions supported by public charity should be conducted on principles of the most rigid economy. The very servants of such institutions should feel bound in honour to regard waste of every kind as criminal and inhuman. Every penny spent unnecessarily means the curtailment of resources, and the denial of help to some poor creature who may be dying for the want of it. The Newcastle managers are making their hospital free in the interests of the working classes, relying on the manliness and honourable feeling of those classes to stand by them in the critical emergency. We do not doubt that the workmen will respond to this appeal to their manhood, and hoist the flag of self-reliance and self-help with true British alacrity and pluck. "England expects that every man this day will do his duty."

Annuities for a Term of Years.

The Guy's Hospital appeal for £100,000, which seems likely to prove entirely successful, has raised a discussion on the desirability of devoting large donations and special funds to the purchase of annuities for a term of years. In the case of Guy's Hospital, it was pointed out that £100,000 would purchase a substantial annuity for ten years, which would amply suffice to increase the income to a sum quite adequate to meet the claims upon the hospital exchequer, if all the beds were occupied. During the ten years of grace thus secured to the managers of Guy's Hospital, means could be easily devised by which the funded property could be placed upon the best possible basis for securing a maximum income, and if it was found to be insufficient for the purpose, then the large firms in the Borough, who have heretofore had every benefit from Guy's Hospital without the privilege of contributing anything to its support, might be invited to subscribe annually to the funds in proportion to the number of cases received at the Hospital from their works, and the actual monetary deficiency which had to be provided for by the Governors. The late Mr. Boys left a considerable sum to the hospitals, with instructions that it should be devoted in each case to the purchase of a ten years' annuity. Many hospitals have received upwards of £600 per annum from Boys' annuity, which expired in most cases at the beginning of the present year. Several of these charities are making special efforts to replace the loss to income thus created, and it would be well if benevolent donors who contemplate helping the hospitals largely in this year of Jubilee, were to follow Mr. Boys' example, and give their money for the purchase of a terminable annuity. We are strongly impressed by the feeling, that it would be well for all voluntary charities if their invested funds never exceeded a maximum of five years' income, for then the managers would be compelled to rely upon the support of the public year by year, to obtain which they must conduct their institutions upon principles which command public confidence and gratitude.

Chemists and Druggists.

The amendment of the laws relating to chemists and druggists is a step in the right direction, but it might have been two or three steps without any disadvantage. Chemists and druggists are the custodians of deadly poisons in every form. The subtlest, the most concentrated, and the most powerful substances in existence are in their charge, to be dispensed to all the world under certain conditions. It is rather an appalling consideration that the shops containing these potent wares are sometimes left in charge of mere youths, who do not know even the Latin names of some of their goods, much less their physical aspect and active properties. It is therefore of the utmost importance that a thoroughly practical knowledge of the appearance and properties of all dangerous substances should be possessed by persons empowered by law to sell them, and heavy penalties should be exacted from those who contravene such necessary statutes. The mere pretender to knowledge should be rigorously shut out of the chemist's occupation. All chemists will agree to this. Will they also agree that the chemist who professes to know and practise the doctor's art should also be duly punished? Perhaps not. Nevertheless, chemists who practise medicine are a very numerous class, and a further amendment of the law which would put a stop to their proceedings is eminently desirable.

